# Effects of cell seeding density on real-time monitoring of anti-proliferative effects of transient gene silencing

**DOI:** 10.1186/s40709-016-0057-4

**Published:** 2016-12-01

**Authors:** Cigdem Selli, Yasemin Erac, Metiner Tosun

**Affiliations:** 1 Applied Bioinformatics of Cancer, Edinburgh Cancer Research Centre, Institute of Genetics and Molecular Medicine, The University of Edinburgh, Crewe Road South, Edinburgh, EH4 2XR UK; 2 Department of Pharmacology, Faculty of Pharmacy, Ege University, 35040 Izmir, Turkey; 3 Faculty of Medicine, Izmir University of Economics, 35330 Izmir, Turkey

**Keywords:** A7r5, Cell density, Huh7, Impedance, RTCA

## Abstract

**Background:**

Real-time cellular analysis systems enable impedance-based label-free and dynamic monitoring of various cellular events such as proliferation. In this study, we describe the effects of initial cell seeding density on the anti-proliferative effects of transient gene silencing monitored via real-time cellular analysis. We monitored the real-time changes in proliferation of Huh7 hepatocellular carcinoma and A7r5 vascular smooth muscle cells with different initial seeding densities following transient receptor potential canonical 1 (TRPC1) silencing using xCELLigence system. Huh7 and A7r5 cells were seeded on E-plate 96 at 10,000, 5000, 1250 and 5000, 2500 cells well^−1^, respectively, following silencing vector transfection. The inhibitory effects of transient silencing on cell proliferation monitored every 30 min for 72 h.

**Results:**

TRPC1 silencing did not inhibit the proliferation rates of Huh7 cells at 10,000 cells well^−1^ seeding density. However, a significant anti-proliferative effect was observed at 1250 cells well^−1^ density at each time point throughout 72 h. Furthermore, significant inhibitory effects on A7r5 proliferation were observed at both 5000 and 2500 cells well^−1^ for 72 h.

**Conclusions:**

Data suggest that the effects of transient silencing on cell proliferation differ depending on the initial cell seeding density. While high seeding densities mask the significant changes in proliferation, the inhibitory effects of silencing become apparent at lower seeding densities as the entry into log phase is delayed. Using the optimal initial seeding density is crucial when studying the effects of transient gene silencing. In addition, the results suggest that TRPC1 may contribute to proliferation and phenotypic switching of vascular smooth muscle cells.

## Background

Impedance-based real-time cellular analysis (RTCA) systems enable label-free, non-invasive and kinetic monitoring of cellular events in contrast to labour-intensive label-based end-point measurements [1]. The xCELLigence RTCA system, developed by Roche Applied Science (Penzberg, Germany) and currently marketed by the original inventor ACEA Biosciences (San Diego, USA), allows dynamic monitoring of various processes including proliferation, migration and invasion [2]. The system is also a powerful and reliable drug discovery tool for toxicological and pharmacological studies [3] including cardiovascular safety testing [4–7] and drug screening [8–11]. Although xCELLigence system is originally designed to work with adherent cells, successful monitoring of non-adherent cells such as cells from haematological malignancies by pre-coating of the cell culture surface with specific adhesive substrates [12] has expanded its area of use.

Since each cell type has its own characteristic growth pattern, the optimum seeding concentration of each cell type gives a short lag period and early onset of logarithmic growth should be determined before the proliferation assays using RTCA system. For this purpose, performing preliminary experiments to obtain the cell growth patterns at different seeding densities is suggested (xCELLigence Application Note No.7/January 2009). We proposed that the optimum cell seeding density also depends on each experimental condition and the actual experiments should also be performed at different seeding densities in addition to the preliminary experiments, especially for transiently-silenced cells.

Following its discovery in mammalian cells [13], RNA interference has been used as a powerful tool to study gene function by administration of small interfering RNA (siRNA) and short hairpin RNA (shRNA) [14]. A significant disadvantage of siRNA application is that its concentration becomes diluted as the cells divide resulting in transient silencing. It is possible to generate long-term knockdown of the gene of interest by integration of shRNA into the host genome [15]; however, the creation of a stable shRNA cell line is time-consuming and may take months. Since stable knockdown requires longer subculturing process, it was not applicable to A7r5 cells because subculturing leads to alterations in A7r5 cell phenotype and proliferation [16]. Therefore, in the present study, we performed transient silencing of transient receptor potential canonical 1 (TRPC1) levels in A7r5 cells.

Although still controversial, TRPC1 has been suggested to be an essential component of store-operated Ca^2+^ (SOC) entry channels in heteromultimeric combinations with other TRPCs [17, 18]. SOC entry, a mechanism activated by emptying of intracellular Ca^2+^ stores [19], was suggested to maintain optimal sarcoendoplasmic reticulum Ca^2+^ levels mediating Ca^2+^ signalling-related cellular processes including proliferation [20]. We previously demonstrated reciprocal changes in TRPC1 and TRPC6 levels in A7r5 vascular smooth muscle cells [21] and in aging rat thoracic aorta [22]. Furthermore, downregulation of TRPC1 significantly elevated SOC entry suggesting the regulatory role of TRPC1 both in A7r5 [21] and Huh7 hepatocellular carcinoma cells [23]. *TRPC1* silencing also suppresses Huh7 cell proliferation without affecting cell migration in real-time cellular analyses suggesting the role of TRPC1 in the regulation of hepatocellular carcinoma cell proliferation [23].

Based on these data, we monitored the real-time changes in proliferation of Huh7 and A7r5 cells with different seeding densities following transient *TRPC1* gene silencing using E-plate 96 and xCELLigence MP system.

## Methods

### Cell culture

Human hepatocellular carcinoma cell line, Huh7, cultured in DMEM (Biological Industries, Cromwell, USA) supplemented with 10% fetal bovine serum (FBS, HyClone, Logan, USA), 2 mM l-glutamine (HyClone, Logan, USA) and 0.1 mM non-essential amino acid solution (Gibco, Waltham, USA). Vascular smooth muscle cell line (A7r5, an immortalized line derived from embryonic rat aorta) cultured in DMEM/Ham’s F12 (Gibco, Waltham, USA) supplemented with 10% FBS (Gibco, Waltham, USA) and 2 mM l-glutamine (Gibco, Waltham, USA). Cells were maintained in a humidified incubator at 37 °C and 5% CO_2_ and were subcultured using 0.5% trypsin–EDTA when reached 70% confluency. Huh7 and A7r5 cells were subcultured with 1:2 and 1:3–1:4 split ratios, respectively, and passage numbers (P#) were recorded. Regular checks for mycoplasma contamination were performed using MycoAlert Mycoplasma Detection kit (Lonza, Basel, Switzerland). After freezing in feeding medium with 5% DMSO, cells were stored in the vapour phase of liquid nitrogen. A7r5 cell line purchased at P# 11 from the American Type Culture Collection (ATCC; CRL-1444). Huh7 cells, originally from Jack Wands Laboratory at Massachusetts General Hospital, Boston, MA, were kindly provided by Professor Mehmet Ozturk (Dokuz Eylul University, Izmir, Turkey), considered to be at their first passage (P# 1) at the time of arrival to our laboratory and were tested for authenticity in 2010.

### Real-time monitoring of proliferation

Real-time monitoring of cell proliferation performed using xCELLigence MP system. E-plate 96, used with xCELLigence system, is a single-use 96 well cell culture plate with bottom surfaces covered with microelectrode sensors (0.2 cm^2^ well surface area; 243 ± 5 µl maximum volume). Real-time changes in electrical impedance measured using the gold microelectrodes and expressed as “cell index” defined as (Rn-Rb)/15, where Rb is the background impedance and Rn is the impedance of the well with cells. Negative control groups (wells containing 200 µl culture medium without cells with cell index values around 0) were tested in every experiment; however, they were not shown in figures in order to simplify the representations.

Before seeding cells into E-plate 96, the background impedance was measured after the addition of 100 µl medium and a 30 min-incubation period at room temperature. Cell density was determined by using a haemocytometer after methylene blue staining. Following the seeding of the appropriate number of cells into the wells, the plate incubated at room temperature for 30 min in order to allow cell settling. Cell proliferation monitored every 30 min for 72 h. Cells were fed with 200 µl well^−1^ fresh medium every 48 h.

### Transient TRPC1 gene silencing

In silencing experiments, Huh7 cells were transfected in 6-well plates with pSUPERIOR.*shTRPC1*, or empty vector as negative control, as described previously [23]. Briefly, to construct pSUPERIOR.*shTRPC1* vector, *TRPC1* silencing shRNA sequence (*shTRPC1*, with a 19 nucleotide-silencing sequence which targets 361–379th nucleotides of TRPC1 mRNA) purchased and cloned into pSUPERIOR.retro.neo + gfp vector (Oligoengine, Seattle, USA). After 48 h vector incubation, Huh7 cells were seeded into E-plate 96 at different densities (10,000, 5000 and 1250 cells well^−1^).

In addition, A7r5 cells transfected with 2 µg pSUPERIOR.*shTRPC1* or empty vector as negative control via 6 µl FugeneHD transfection reagent (Roche Applied Science, Penzberg, Germany) were seeded into E-plate 96 at different densities (5000 and 2500 cells well^−1^) 48 h after the vector incubation.

### Quantitative real-time RT-PCR

Effects of vector transfection on TRPC1 expression levels in A7r5 cells were measured by quantitative real-time RT-PCR using FastStart DNA Master SYBR Green I kit and LightCycler 1.5 (Roche Applied Science, Penzberg, Germany). High Pure RNA Isolation Kit (Roche Applied Science, Penzberg, Germany) and Dynamo cDNA Synthesis Kit (Finnzymes, Waltham, USA) used to perform total RNA isolation and reverse transcription, respectively. Primers used for TRPC1 (NM_053558) and beta-actin (NM_031144) were as follows: forward 5ʹTGGTATGAAGGGTTGGAAGACʹ3 [24], reverse 5ʹTGCTGTTCACAGAAGATGCCʹ3 [25], and forward 5ʹAGTGTGACGTTGACATCCGTʹ3 [26], reverse 5ʹGACTCATCGTACTCCTGCTTʹ3 [26]. TRPC1 expression levels were normalized to that of internal β-actin and expressed as [TRPC1/β-actin × 100].

### Data analysis

Data expressed as mean ± standard deviation. “n” represents the number of samples. Statistical significance between the means of two groups was evaluated using Student’s *t* test, with *p* < 0.05 considered significant. Data analyses and graphical presentations performed using GraphPad Prism 5 (La Jolla, USA).

## Results

### Effects of cell seeding density on Huh7 proliferation following TRPC1 silencing

**Fig. 1:**
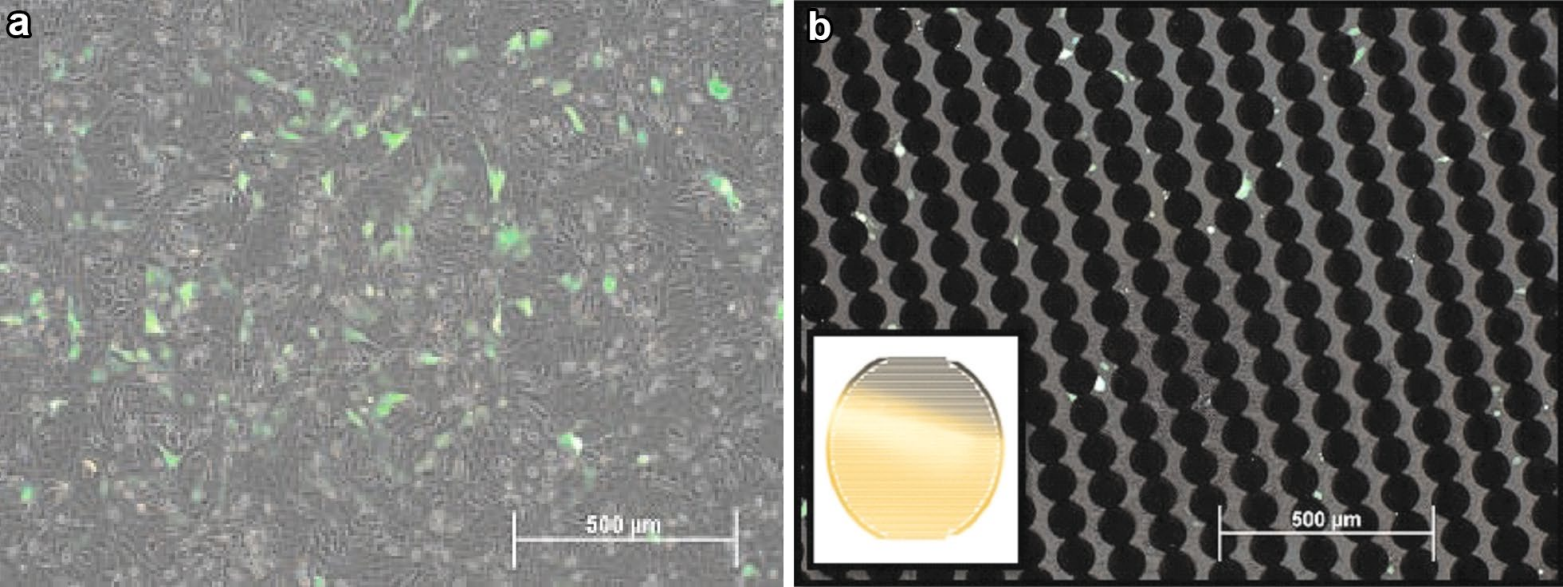
Fluorescent microscopy images of Huh7 cells. **a** Silencing vector-transfected Huh7 cells were observed in a 6-well plate after 48 h incubation before seeding into E-plate 96. **b** At the end of the proliferation assay, Huh7 cells on E-plate 96 were visible between microelectrodes seen as aligned *closed circles*. The *insert* represents the gold microelectrode sensors covering the *bottom surface* of the well (from the manufacturer’s manual)

In order to determine the effects of seeding density on anti-proliferative effects of transient gene silencing, real-time changes in proliferation were monitored in *TRPC1*-silenced Huh7 cells seeded at different densities into E-plate 96. Huh7 cells transfected with silencing vector that also emit GFP signal was observed using an inverted fluorescent microscope (Olympus IX71) and the transfection efficiency was determined (70%) by monitoring GFP fluorescence emission at 520 nm with excitation at 460–490 nm (Fig. 1a). Huh7 cells were also observed on E-plate 96 at the end of each experiment. Although the non-transparent gold microelectrodes preclude the accurate prediction of the transfection efficiency, an apparent decrease in fluorescence signal was monitored due to dilution of transfected cells as the cells divide, as expected (Fig. 1b). The company (ACEA Biosciences, San Diego, USA) is currently marketing modified version of plates with a small opening in the electrode array, E-Plate VIEW, allowing the visual inspection the cells under an inverted microscope.

**Fig. 2:**
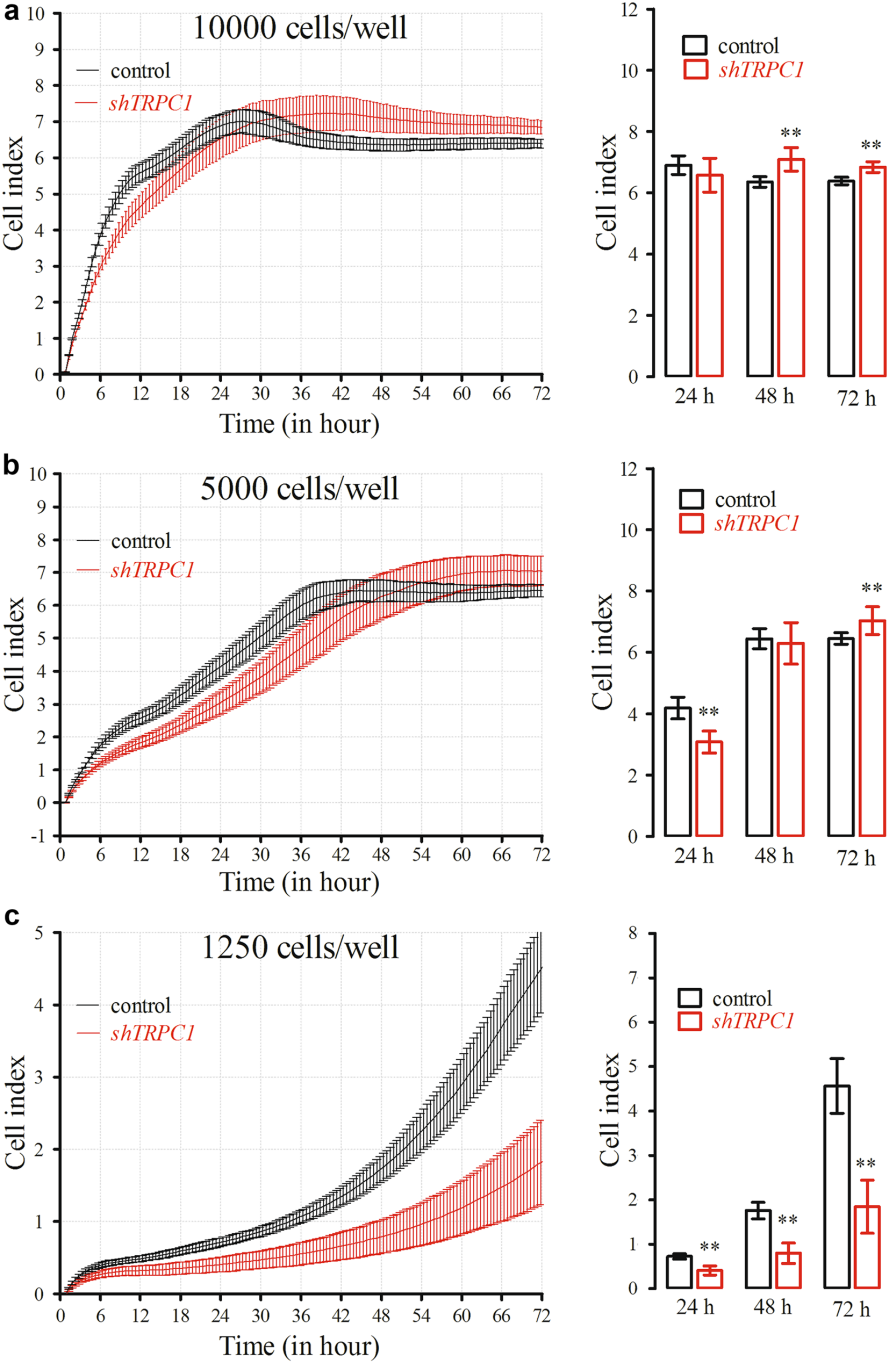
Real-time proliferation of *TRPC1*-silenced Huh7 (P# 26) cells at different densities. At 48 h following shTRPC1 transfection, cells were seeded on E-plate 96 and cell proliferation monitored real-time for 3 days. The proliferation curves and cumulative data of cells at different densities (cells well^−1^); **a** 10,000, **b** 5000 and **c** 1250 are shown (**p < 0.01, n = 10, n = 9 and n = 12, respectively)

*TRPC1* silencing did not inhibit the proliferation of Huh7 cells at 10,000 cells well^−1^ seeding density (n = 10, Fig. 2a). When seeded at 5000 cells well^−1^, proliferation was significantly suppressed for the first 24 h (*p* < 0.01; n = 9; Fig. 2b). Both control and silenced-cells reached plateau cell index after 30 h at 10,000 cells well^−1^ seeding density. At 5000 cells well^−1^ density, control and silenced-cells reached plateau cell index after 36 and 42 h, respectively. After these steady-state time points, significant but incoherent increases in proliferation were observed at both seeding densities (*p* < 0.01; Fig. 2a, b). However, *TRPC1* silencing significantly inhibited the proliferation of Huh7 cells compared to control cells in 1250 cells well^−1^ seeding density at each time point for 72 h (*p* < 0.01; n = 12; Fig. 2c).

### Effects of cell seeding density on A7r5 proliferation following TRPC1 silencing

**Fig. 3:**
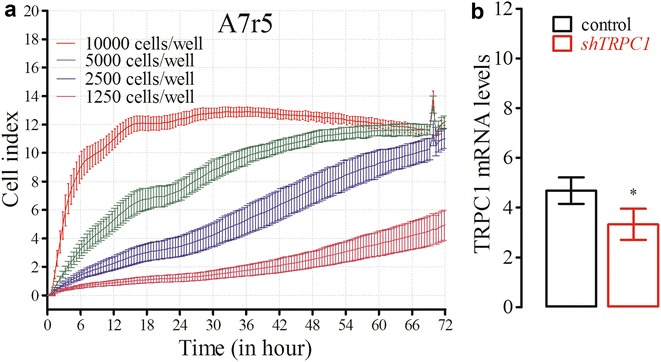
Real-time proliferation curves and cumulative mRNA expression data of A7r5 cells. The proliferation of A7r5 cells (P# 17) at different cell densities were monitored for 72 h (**a**; n = 8). TRPC1 mRNA expression levels were determined 72 h following silencing vector transfection (**b**, *p < 0.05, n = 3)

A7r5 proliferation curves at four different seeding densities (10,000–1250 cells well^−1^) were monitored before determining the effects of silencing on proliferation. Cell index increased proportionally to A7r5 cell densities as expected (Fig. 3a). Cells reached a plateau after 24 and 48 h at 10,000 and 5000 cells well^−1^ densities, respectively. Cells at 2500 and 1250 cells well^−1^ seeding densities showed exponential increases in proliferation and did not reach the plateau after 72 h.

The effects of *TRPC1* silencing on expression levels were determined using real-time qRT-PCR before performing the proliferation assay. *TRPC1* silencing significantly inhibited the TRPC1 mRNA expression levels compared to control cells (*p* < 0.05, n = 3, Fig. 3b). Although the silencing partially (29%) inhibited TRPC1 mRNA levels, its inhibitory effect on cell proliferation was significant.

**Fig. 4:**
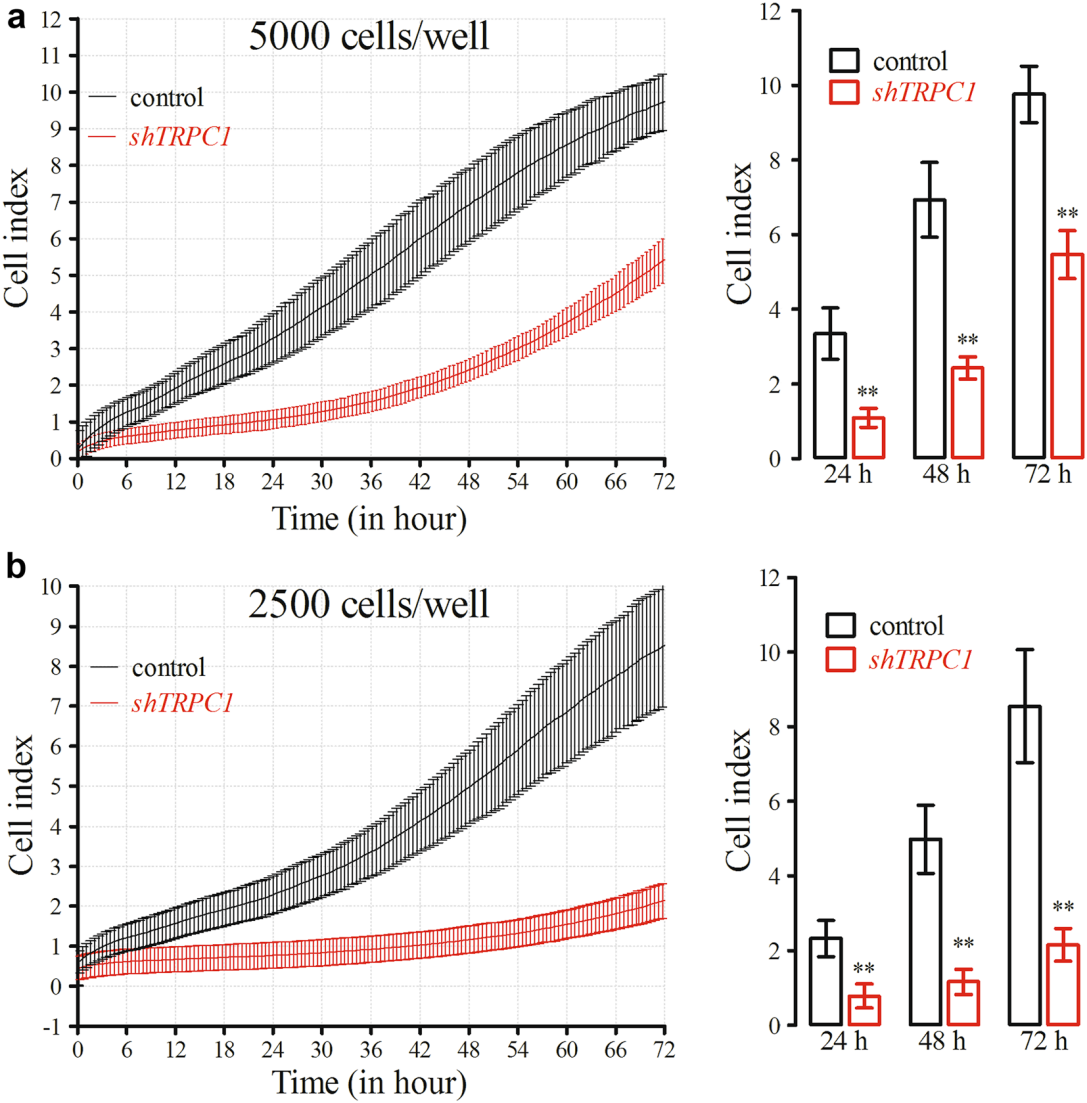
Real-time proliferation of *TRPC1*-silenced A7r5 cells (P# 20) at different cell densities. At 48 h following shTRPC1 transfection, 5000 (**a**) and 2500 (**b**) cells well^−1^ were seeded on E-plate 96 and cell proliferation rates were monitored real-time for 3 days (**p < 0.01, n = 8)

Based on the proliferation curves previously performed to determine the optimum cell density (Fig. 3a), real-time changes in proliferation were monitored at 5000 and 2500 cells well^−1^ seeding densities in *TRPC1*-silenced A7r5 cells. *TRPC1* silencing significantly inhibited the proliferation of A7r5 cells at both 5000 and 2500 cells well^−1^ seeding densities for 72 h (*p* < 0.01, n = 8, Fig. 4). Both control and silenced cells did not reach the plateau cell index at either seeding densities.

## Discussion

Since the initial seeding density is critical in functional tissue engineering, the proliferation of human umbilical cord mesenchymal stem cells (hUCMSCs) seeded on calcium phosphate cement, a scaffold material used for bone tissue engineering, at different densities has been investigated previously [27]. Zhou et al. showed that hUCMSC proliferation and osteodifferentiation increased proportional to cell seeding density from 50,000 to the optimum value of 300,000 (cells in a 24-well plate), with a decrease over this limit [27]. Furthermore, to determine the endothelial cell-biomaterial interaction that had an impact on the development of biomedical implants, the effects of cell-seeding density on the proliferation rate of human umbilical vein endothelial cells (HUVEC) seeded on different biomaterials including tissue culture polystyrene were investigated [28]. Maximal HUVEC proliferation was obtained at an initial seeding density of 1000 cells cm^−2^ with a sharp decrease both above and below of this particular density [28].

Although cell proliferation is required for physiological processes such as renewal of intestinal epithelium [29] and wound healing [30], its abnormalities are associated with various diseases such as tumorigenesis [31]. The involvement of TRPC1 in cell proliferation in different types of cancers [32–34] as well as in endothelial progenitor cells [35] has been previously reported. In addition, Huh7 cell line was shown to have a subpopulation of cells with hepatic cancer stem cell-like properties that express alpha-fetoprotein and epithelial cell adhesion molecule (EPCAM), a hepatic stem cell biomarker [32].

In our previous study, inhibitory effects of silencing vector administration on TRPC1 expression levels in Huh7 cells were determined by quantitative real-time RT-PCR and western blot analyses [23]. After *TRPC1* silencing vector transfection, mRNA levels were shown to be inhibited reversibly with a significant decrease at 48 h and recovered at 72 h [23]. Furthermore, we observed significant suppression in proliferation rates and increase in doubling time in *TRPC1*-silenced Huh7 cells at 2500 cells well^−1^ seeding density [23]. Based on these data, among the hepatocellular carcinoma cell lines, Huh7 cells were chosen to further study the effects of cell seeding density on the proliferation of *TRPC1*-silenced cells. Inhibitory effects of transient *TRPC1* silencing on Huh7 proliferation rates were masked at 10,000 cells well^−1^ seeding density with a significant decrease only after 24 h at 5000 cells well^−1^ seeding density. Moreover, the effects of silencing were much more significant at 1250 cells well^−1^ seeding density at each time point throughout the whole assay suggesting the monitoring cells in very early points of log period as well as before reaching the plateau phase is essential to detect the effects of transient silencing. Therefore, the seeding density that allows cells to reach the plateau levels after 72 h or later should be used for Huh7 cells.

In addition to cancer cells, we also monitored the effects of seeding density on the proliferation of *TRPC1*-silenced A7r5 cells which has a significance in examining the vascular contractile and proliferative phenotypes in vitro [36]. It is known that vascular smooth muscle cells have the ability of plasticity with a wide range of phenotypes besides their primary contractile phenotype [37]. Evidence suggests that switching from contractile to proliferating (non-contractile/synthetic) phenotype is associated with vascular diseases [38, 39]. Differential expression of calcium handling proteins including upregulated TRPC1 and TRPC6 expression levels were associated with the proliferative phenotype [39, 40]. In our previous study investigating the effects of A7r5 passaging that may mimic the phenotypic switching of vascular smooth muscle cells, we observed upregulated SERCA2b mRNA and SOC entry levels along with suppression of proliferation [16]. In the present study, knockdown of TRPC1 inhibited A7r5 cell proliferation suggesting its possible contribution to vascular smooth muscle cell proliferation and phenotypic switching. Furthermore, the inhibitory effect of transient *TRPC1* silencing on A7r5 proliferation was observed at both seeding densities tested in our study (5000 and 2500 cells well^−1^, respectively) suggesting that the seeding density that allows cells to reach the plateau levels after 72 h or later is optimal for studying the effects of transient silencing on A7r5 cells.

In silencing experiments, at least 72 h incubation is required to observe the suppression of target protein and resulting phenotypic alterations. We have previously shown that TRPC1 mRNA levels were inhibited reversibly with a significant decrease at 48 h and recovered at 72 h in Huh7 cells [23]. In addition, TRPC1 protein levels decreased significantly at 72 h after silencing vector transfection in A7r5 and Huh7 cells [23]. In the current study, cells were seeded on E-plate 96 after 48 h vector incubation and therefore, at least a 24-h period is required to observe an effect on protein levels and proliferation. When cells were seeded in higher densities, they reached plateau proliferation levels at 24 h which may limit the production of siRNA. Significant changes in proliferation were observed if cells were seeded in optimum densities possibly allowing sufficient time to downregulate target protein levels. Determination of mRNA and protein levels 24 h following seeding on E-plates at different densities requires further investigation.

## Conclusions

The effects of transient silencing on cell proliferation vary depending on the cell seeding density at the start of the RTCA experiments. Higher seeding densities mask the significant changes in proliferation rates whereas they become clear at lower seeding densities as the log phase is delayed. Therefore, determination of optimal cell seeding density for real-time monitoring of proliferation in transiently-silenced cells is crucial for more accurate data acquisition and evaluation. We suggest that real-time monitoring studies should be performed using a range of cell type specific initial seeding densities in order to determine the effects of transient gene-knockdown. This would help to improve our knowledge about the outcome of transient gene silencing. Beyond the technical aspects, the data suggest that TRPC1 may contribute to proliferation and phenotypic switching of vascular smooth muscle cells.
